# A call for a moratorium on the .health generic top-level domain: preventing the commercialization and exclusive control of online health information

**DOI:** 10.1186/s12992-014-0062-z

**Published:** 2014-09-26

**Authors:** Tim K Mackey, Gunther Eysenbach, Bryan A Liang, Jillian C Kohler, Antoine Geissbuhler, Amir Attaran

**Affiliations:** Global Health Policy Institute, 8950 Villa La Jolla Drive, Suite #A204, La Jolla, CA 92037 USA; Department of Anesthesiology and Division of Global Public Health, University of California San Diego School of Medicine, 200 W Arbor Drive, San Diego, CA 92103-8770 USA; Institute for Health Policy, Management, and Evaluation, University of Toronto, 27 King’s College Cir, Toronto, ON M5S Canada; Centre for Global eHealth Innovation and Techna Institute, University Health Network, 190 Elizabeth Street, Toronto, M5G 2C4 Canada; Leslie Dan Faculty of Pharmacy, University of Toronto, 27 King’s College Cir, Toronto, ON M5S Canada; University of Geneva, Geneva, Switzerland; Health On the Net Foundation, Chêne-Bourg, 1225 Switzerland; International Medical Informatics Association, CH-1225 Chêne-Bourg, Geneva, Switzerland; Faculties of Law and Medicine, University of Ottawa, One Stewart Street, Ottawa, ON K1N 6 N5 Canada

**Keywords:** eHealth, Global health governance, Information technology, Internet, Domain names, Health Internet

## Abstract

In just a few weeks, the Internet could be expanded to include a new .health generic top-level domain name run by a for-profit company with virtually no public health credentials - unless the international community intervenes immediately. This matters to the future of global public health as the “Health Internet” has begun to emerge as the predominant source of health information for consumers and patients. Despite this increasing use and reliance on online health information that may have inadequate quality or reliability, the Internet Corporation for Assigned Names and Numbers (ICANN) recently announced it intends to move forward with an auction to award the exclusive, 10 year rights to the .health generic top-level domain name. This decision is being made over the protests of the World Medical Association, World Health Organization, and other stakeholders, who have called for a suspension or delay until key questions can be resolved. However, rather than engage in constructive dialogue with the public health community over its concerns, ICANN chose the International Chamber of Commerce—a business lobbying group for industries to adjudicate the .health concerns. This has resulted in a rejection of challenges filed by ICANN’s own independent watchdog and others, such that ICANN’s Board decided in June 2014 that there are “no noted objections to move forward” in auctioning the .health generic top-level domain name to the highest bidder before the end of the year. This follows ICANN’s award of several other health-related generic top-level domain names that have been unsuccessfully contested. In response, we call for an immediate moratorium/suspension of the ICANN award/auction process in order to provide the international public health community time to ensure the proper management and governance of health information online.

## Background

An international debate that may very well determine the future of online health information is currently underway, yet prospects for the global public health community and consumer protection advocates appear bleak following recent decisions by the Internet Corporation for Assigned Names and Numbers (“ICANN”) to proceed forward with the auction of a new .health top-level domain over the objections of numerous public health stakeholders [1]. At stake is the future of the “Health Internet”, a term used by the World Health Organization (“WHO”) to describe the wide range of benefits *and* risks the Internet now poses to individual consumers, health professionals, and local, national, and global public health systems [2].

Wikipedia, the world’s largest reference website and a product of the modern digital age, aptly describes the Internet as a global system of interconnected computer networks that connects billions of devices worldwide, many of which now predominantly feature and deliver health information and services (e.g., websites, social media platforms, mHealth applications, connected devices) [3]. In order to ensure that global users can navigate and understand this vast network of resources, the Internet uses unique, hierarchical identifiers to organize the global Internet name space into familiar constructs like website names and e-mail addresses [4]. The top-level domains (“TLD”) are the highest level of this naming system consisting of names in the root zone, or simply everything after the final dot in a web address (i.e*.,http://www.example.[COM]*). Just below TLDs are the second-level domains (“SLD”), generally managed by domain name registrars (i.e*.,http://www.[EXAMPLE].com*). For the most part, anyone who has owned a website URL at some time has bought its name from a domain name registrar.

The entity that manages this hierarchical naming system and the roughly 500 accredited domain name registrars is ICANN, a California non-governmental organization established by the US government, but notionally independent of it [4,5]. ICANN relies on an international Board of Directors consisting of various ICANN constituents, a CEO, staff, and advisory committees consisting of stakeholders from national governments, Internet technical experts, and Internet organizations to inform its decisions [4].

Since the Internet is not a single entity, but a voluntary federation of networks, it requires a leviathan such as ICANN and its affiliates (specifically The Internet Assisted Numbers Authority) to impose certain standards on the namespace and to accredit domain name registrars [5]. Because it governs the majority of the domain name system, ICANN bears great responsibility for those standards, and how the Internet can be used to help or harm individual users.

However, ICANN’s governance structure has been criticized for its lack of adequate stakeholder participation and insufficient transparency bringing into question the appropriateness of its role in newly-emerging Internet governance [4]. For example, under ICANN’s Uniform Domain Name Dispute Resolution Policy, domain name owners are forbidden from “registering [a] domain name for an unlawful purpose”, and prohibited from “knowingly [using] the domain name in violation of any applicable laws or regulations” [6]. These reasonable obligations, however, lack enforceability, because ICANN has no appeal process or mechanism to take *proactive* action against websites that are being used for unlawful purposes or that violate laws that accredited registrars fail to report [7]. Consequently, many websites feature illicit online content with clear public health and patient safety concerns that registrars take no action against, such as websites selling medicines without a prescription and that also potentially traffic counterfeit or falsified medicines [7-12]. These websites are connected with organized criminal enterprises and have caused injury and even death, such as the case of USA teenager Ryan Haight, who died from an overdose of prescription opioid drugs illegally purchased from an online pharmacy [8,9].

Because ICANN’s processes have not been sufficient to address current threats to the safety and security of the Health Internet, there is reason to worry that the situation will be exacerbated by simply awarding the new health domains to the highest bidder (explained below). ICANN’s processes appear to favor business interests and generation of profits over the future integrity of the Health Internet, failing to make any tangible commitments to protect public health or enforce norms as would be found in a responsible global governance framework [4,11,13].

### ICANN’s new gTLD program

Currently, ICANN has an ambitious plan to vastly expand Internet names that was launched in 2011, over and above the 21 TLDs then in existence [13]. The plan calls for creating new “generic top-level domain names” (“gTLDs”), a category of TLDs which can consist of virtually any TLD name suggested by an applicant, including TLDs in different languages/characters, corporate brands, recognized social communities, geographical locations, generally under the broad category of open TLDs (with generally no restrictions and open to the public for registration) [14].

With this expansion, domain names will continue to evolve into a form of intellectual property, as they will carry exclusive use rights for successful registrars, will readily act to identify businesses and/or services, and can be tailored by owners for reasons of branding and identity, as is the case with trademarks. Importantly, this new process of governing the Internet namespace will preclude important decisionmaking processes regarding equitable and safe access of specific gTLDs and their related SLDs, as successful registrars will largely have exclusive control and rights on how to manage their gTLD name space.

The new ICANN gTLD program resulted in 1,930 applications, the majority of which (74.6%, N = 1440) have now passed initial ICANN evaluation [15]. As of the beginning of June 2014, 15.3% (N = 295) of applicants have successfully completed the entire gTLD application process and have had their gTLDs introduced to the Internet, while others are under contractual negotiation and soon to launch [15]. Still others are in the process of contention resolution, which is a process to resolve the award of a gTLD when there is more than one applicant for the same gTLD name, which usually ends in an auction [14].

The current crop of new gTLD applications also includes more than a dozen health-related terms, which depending on how they are used (or abused) could effectively shape the future of health information online, and very likely consumer and patient health behaviors and outcomes [13,16]. Among the new health-related gTLDs are .doctor, .medical, .healthcare, .hospital, and most importantly, .health (see Table 1 for complete listing) The adverse impact of ICANN’s award decisions will emerge when these health-related gTLDs are leased out by ICANN on 10 year, exclusive contracts to whichever domain name registrars are chosen or win an auction, and the domain name registrars begin selling SLDs (i.e., website names) to persons and companies who may seek to maximize profit without due regard to the integrity of online health content [13,16].

**Table 1:** **Health-related gTLD status and proposed safeguards (as of June 2014)**

**gTLD string**	**GAC recommended safeguard (Closed/Open)?**	**In string contention? (Y/N)**	**Current ICANN status**	**Applicant(s)**
.cancerresearch	N/A	No	Pre-delegation testing	**Australian Cancer Research Foundation**
.clinic	Open	No	Delegated	**Donuts (Goose Park, LLC)**
.dental	Open	No	Delegated	**Donuts (Tin Birch, LLC)**
.dentist	Closed	No	Delegated	**Donuts (Outer Lake, LLC)**
.diet	Open	No	In contracting	Famous Four Media (dot Diet Limited)^a^
				**Uniregistry, Corp.**
				Donuts (Pioneer Hill, LLC)^a^
.doctor	Open	Yes	In auction	Donuts (Brice Trail, LLC)
				Radix (DotMedico TLD Inc.)^b^
				The Medical Registry Limited
.fit	Open	No	In contracting	Famous Four Media (Platinum Registry Limited)^a^
				**Top Level Domain Holding**
.fitness	Open	No	Delegated	**Donuts (Brice Orchard, LLC)**
.health	Open	Yes	In auction	DotHealth, LLC
				Afilias
				Donuts (Goose Fest, LLC)
				Famous Four Media (dot Health Limited)^a^
.healthcare	Open	No	In contracting	**Donuts (Silver Glen, LLC)**
.heart	Open	No	Withdrawn	American Heart Association, Inc.^a^
.hiv	Open	No	Delegated	**dotHIV gemeinnütziger e.V.**
.hospital	Closed	No	On-hold^1^	Donuts (Ruby Pike, LLC)^b^
.med	Open	Yes	On-hold^1^	DocCheck AG^a^
				Second Generation Ltd. (Medistry LLC)^b^
				HEXPAS SAS^b^
				Google (Charleston Road
				Registry Inc.)^c^
.medical	Closed	No	On-hold^1^	Donuts (Steel Hill, LLC)^b^
.pharmacy	Closed	No	In contracting	**National Association of Boards of Pharmacy**
.physio	Open	No	Transition to delegation	**PhysBiz Pty Ltd (Glenn Ruscoe)**
.skin	N/A	No	In contracting	**L’Oréal**
.stroke	N/A	No	Withdrawn	American Heart Association, Inc.^a^
.surgery	Closed	No	Delegated	**Donuts (Tin Avenue, LLC)**
.健康 (Chinese equivalent of “healthy”)	Open	No	In contracting	**Stable Tone Limited**

**KEY:**

**Bold:** indicates the successfully awarded applicant or an applicant who is currently in contracting/pre-delegation testing that will likely lead to completion.

**Open:** Proposed as GAC Category 1 strings with safeguards 1–3 applicable. These requirements generally state that operators will include in their Registry-Registrar Agreements provisions for privacy and consumer protection and compliance with applicable laws, but leave TLDs generally Open to registration.

**Closed:** Proposed as GAC Category 1 strings with all safeguards 1–8 applicable. In addition to safeguards 1–3, they would also most importantly require that registry operators work with relevant regulatory or industry self-regulating bodies and include requirements that registrants posses any necessary authorizations, charters, licenses and/or other related credentials for participation.

^1^ICANN Objection determinations where Independent Objector has prevailed on at least one objection (e.g. community or limited public interest).

^a^Withdrawn

^b^On hold – Objection.

^c^Will not proceed.

If this step is taken, then without violating current ICANN rules hypothetically one could begin to see websites with the following URLs as examples [13]:

- *http://www.[smoking].[health](potentially purchased by a tobacco company)*
- *http://www.[vaccinatekids].[health](potentially purchased by anti-vaccine activists)*
- *http://www.[obesity].[health](potentially purchased by a junk food company)*
- *http://www.[cancer].[doctor](potentially purchased by unscrupulous vendors catering to the desperate dying)*

It is self-evidently problematic to propound online scientifically unfounded information, or biased information, in an era where consumers and patients are increasingly using the Internet as their primary source for health information [3,13,17]. Importantly, legal protections of freedom of speech do have limits, especially in the context of use and marketing by corporations, which is why health warnings and/or labeling are already carefully regulated [18]. By creating new, largely un-regulated domain names for vast swathes of health information online, ICANN may be setting a dangerous and potentially wide-ranging precedent that could impede future efforts to establish reliable health information and services online or offline [13,16].

### Health-related gTLD concerns

Despite the growing importance of health information online, little is known about the characteristics of health-related gTLD applicants. ICANN has sought only limited answers from the applicants about which sorts of Internet content they will allow or refuse, who they will let purchase SLDs, whether certain controversial SLD names will be reserved or prevented from being misused, whether conflicts of interest will be disclosed, and how disputes over inappropriate conduct will be adjudicated [13].

What is clear, however, is that several privately held, for-profit businesses, many of which are completely unknown to the public health field and have no such expertise, are actively seeking or have already been awarded these new health domains and propose few if any needed restrictions on future use [13,16]. Some applicants, as in the case of the .health gTLD, have responded to public scrutiny by offering limited application changes, but how their commitments would be implemented and enforced remains unclear, not least because of ICANN’s lack of enforcement of existing rules like the Uniform Domain Name Dispute Resolution Policy [7].

It is difficult to envisage these concerns being properly addressed, given wide-spread debate, concern, and criticism of ICANN’s new gTLD process, particularly in relation to health-related domains [13,16]. For example, a company named Donuts Inc. has applied to ICANN for hundreds of gTLDs, with funding from private equity firms that undoubtedly expect a “healthy” return on their investments [19,20]. Included in these efforts are more than 10 applications that Donuts Inc. filed for health-related gTLDs, including some that are uncontested with no other applicants and that have already been awarded [13]. Once Donuts Inc. acquires these names, then its likely business model will be to generate maximum returns for its shareholders/owners, meaning to sell the maximum number of SLDs at the highest possible price. Such incentives are not easily reconciled with potential concerns regarding the quality of health information they offer, and they would also favor deep-pocketed SLD buyers who could outbid other potential purchasers who might be better suited to propound reliable health information [13,16]. That Donuts Inc. might be less than punctilious may be a valid concern, as consumer and Internet watchdogs have already raised concerns about it being connected with spammers and cybersquatters [20].

### Losing the fight for health domains

Though the stakes for online health information are high, the broader public health or non-profit sectors are paying little attention to ICANN’s unfolding process [6,9]. Indeed, only one non-profit/foundation (the Australian Cancer Research Foundation) is attempting to secure a health gTLD, while another (the American Heart Association) has withdrawn its applications [13]. No doubt part of the reason that the public health and non-profit sector has stayed away is due to ICANN, which although it calls itself non-profit has set the price of gTLDs too high for most: gTLD applications cost an initial US$185,000 and $25,000 annually thereafter, not including the technical infrastructure, expertise, and costs to act as a domain registry operator [13,14].

The few who have been paying attention in the public health or non-profit sectors have been singly opposed to ICANN’s plans for health-related domains [13]. National governments (France and Mali), physicians (the World Medical Association), other professionals (the International Medical Informatics Association), non-governmental organizations (Save the Children), the world’s largest trading bloc (the European Commission), the United Nations (the World Health Organization), and even ICANN’s own ombudsperson (the Independent Objector), have formally lodged objections and expressed serious concerns, yet without persuading ICANN to suspend its plans for assigning the .health gTLD [1,13]. As recently as its meeting of June 6, 2014, ICANN’s Board commented on receiving these expressions of concern, but decided that there were “no noted objections to continuing to move forward with the applications” for .health and other health-related gTLDs [1].

Yet while ICANN has chosen to disregard these cautions, it has favored applicants from other sectors including larger corporations. As an example, when ICANN’s Governmental Advisory Committee protested that certain new gTLDs (.wtf, .fail, .gripe and .sucks) could be wielded against businesses by angry customers – no corporation ever wants to see *http://www.[YOURBRANDNAME].sucks* – ICANN singled these out for “special safeguards” and suspended further plans [16,21]. No such reprieve or moratorium was ever granted for the health-related gTLDs, although ICANN agreed that a few could possibly have closed entry requirements (i.e., only open to registrants demonstrating a certain status or credential such as healthcare licensure), while most (including .health) would have open registration (see Table 1) [21].

However, even the proposed “closed” safeguards are not specific to public health, and new gTLDs such .casino, .creditcard, and .poker are in the same safeguards group as .hospital, .doctor, .pharmacy, .dentist, .surgery, and so on. Additionally, the contested .health gTLD has fewer safeguards still: only a “requirement to comply with all applicable laws” (which should always be the case), and a vague, undefined obligation “to implement reasonable and appropriate security measures … [for] sensitive health … data” (which also is the law and a given in many countries) [21]. Hence ICANN’s procedural decisions have not afforded any special safeguards to health-related domains, even though it has extended protections for arguably more trivial gTLDs, such as .wtf [13].

Beyond ICANN’s policies, the quality of the companies applying to acquire exclusive rights to .health have raised concerns. Our anxieties about ICANN’s plans for the .health and the prospective applicants are by no means unique. ICANN’s ombudsperson, the Independent Objector, lodged an official complaint that mirrors several of our critiques [22,23]. ICANN referred this objection to the International Chamber of Commerce (“ICC”) to adjudicate, although ICC has no public health expertise and obvious conflicts of interest (e.g., several tobacco companies are members) [24]. Perhaps unsurprisingly, the ICC ruled in favor of all existing .health business applicants (including Donuts Inc. and Dot Health LLC.), effectively continuing the award process that will end in the contracting of .health to the highest bidder [13]. Though the ICC acknowledged that health and access to health information is different from other commodities, it made the egregious error of omitting the fact that treaties such as the *International Covenant on Economic, Social and Cultural Rights* has established the fundamental human right to health in international law [25]. “Assuming” that such a right exists, the ICC adjudicators wrote, it was not clear “that the operation of .health gTLD registry by a private entity would inhibit or impair the access to accurate health information” [22]. ICC also rejected placing *any* conditions on the applicant to provide additional safeguards in response to concerns raised by the Independent Objector [22,23].

More recently, at the beginning of June 2014, ICANN’s New gTLD Program Committee (a Committee granted Board-level authority to make decisions on the new gTLD program) stated that it had reviewed requests from the international community requesting additional safeguards – but decided unanimously to continue to move forward with the .health and health-related applications [1]. Hence, as a result of ICANN’s prior and continued inattention to public health interests, as of the beginning of June 2014, of the 19 active health-related gTLDs, eight (42.1%) have either already been successfully delegated to private operators, or are in the course being delegated (Table 1). An additional six applications are currently in the contracting phase with ICANN, meaning close to three-quarters (73.7%, N = 14) of all health-related gTLD applications have already been awarded and/or will become active shortly. Alarmingly, Donuts Inc. is the successful applicant for six of these awarded applications. Additionally, two gTLDs (.doctor and .health) are currently scheduled for auction as they have multiple applicants. Only 15.8% (N = 3) of all health-related gTLD applications are on hold as they have had at least one successful objection that has prevailed through ICANN’s dispute process. Overall, the rush of ICANN to put health-related gTLDs on the market has mostly prevailed, regardless of the potential consequences.

### Arguments by .health applicants

In response to public scrutiny, the .health gTLD applicants have countered that there is no way to determine what constitutes “quality” health information; for this reason, they argue, they should not be held accountable to any standards [13]. They also argue there are no internationally-recognized principles/standards relating to the word “health” that require protection under international legal norms; that proper safeguards are already in place; and that they will require registrants to follow generally accepted legal norms and “applicable law” [22].

However, these arguments are simply focused upon minimum if any legal compliance and are not grounded in public health interests [13,16]. Following generally accepted legal norms is not a meaningful safeguard because it merely restates what any entity would be required to do in its operations, regardless of owning a gTLD. Further, as previously stated, enforcement mechanism in ICANN have been criticized and may therefore be without sufficient consequence—as with ICANN’s failure in its ability to enforce the Uniform Domain Name Dispute Resolution Policy on Internet pharmacies [7]. True safeguards must be enforceable and focused on protecting the user, especially where national law is absent, as is the case of several countries that fail to or do not adequately regulate pharmaceutical promotion [11,26].

Legal compliance and reliance upon “applicable law” is also dependent upon the development of important online health-related legal frameworks from local, national, regional, and the international level that are still in the process of maturation. This includes lack of legislation addressing cybersecurity issues such as educating users about Internet safety and literacy and the regulation of illicit online pharmacies [27]. According to a WHO report, only 47% had government-sponsored websites/initiatives for information and education of Internet safety and literacy and 66% had *no* legislation regulating Internet pharmacies, with countries enacting legal protections highly skewed towards higher-income countries [27]. Most importantly, 55% of responding countries reported that voluntary compliance by content providers/websites was the method for establishing quality criteria for health-related sites [27].

Nor is the postmodern relativism that there is no way to discern “quality” health information from inaccurate or misleading information a sufficient conclusion to address the issue: if that were the case then the fundamental processes of the scientific community (such as peer-review, evidenced-based decision making, expert consensus, and/or randomized clinical trials methods) to ensure a certain level of validity of health information that may be presented and relied upon by consumers and clinicians would be viewed as unnecessary or unimportant as well. While efforts to improve quality of online health information are presently limited, the task is not new or infeasible. Initiatives such as MedCIRCLE (an international project funded by the EU to collaborative ratings and use of metadata for enhancing transparency and identification of Internet health information) and the HealthOntheNetFoundation Code (a voluntary ethics code and certification system aimed at encouraging dissemination of quality Internet health information in place since 1996) are expressly aimed at improving the quality of online health data as well as helping consumers identify sites that have reliable and certified information/sources [28-32].

### Potential consequences

ICANN’s gTLD contracts last a decade and contain a presumption of indefinite renewability. Consequently, the public health community must address this issue appropriately now to avoid potentially significant long-term negative consequences. As an example of a failure to monitor health-related marketing, fraud and abuse in pharmaceutical promotion has been well documented and has led to record-breaking criminal and civil fines in countries such as the USA, even where regulation/oversight exists [18,33]. Direct-to-consumer advertising (“DTCA”) of pharmaceuticals has also been criticized as leading to higher national healthcare costs due to overutilization of expensive drugs, misrepresentation of risks versus benefits, and has been associated with drugs with high-risk profiles [34,35]. It is already worrisome that, although DTCA is only allowed in the USA and New Zealand among developed countries, it is transmitted across geopolitical borders via the Internet where the jurisdiction of drug regulatory bodies is lacking [10,11,36]. Expanding opportunities for unregulated pharmaceutical DTCA through health-related gTLDs could likely make this situation worse.

There is also the risk that organized criminals could use the new gTLDs for online health fraud and abuse. Potential and current threats to the security and safety of the Health Internet include: platforms of health-related email spam, illicit online pharmacies, fraudulent solicitation of personal health information, fraudulent solicitation of funds for health services, etc. [11,27]. Hence, without appropriate oversight, the new gTLDs could represent a conduit for various types of deceptive, fraudulent or illegal activity that could directly harm consumers because of the absence of effective ICANN enforcement mechanisms and the limits of jurisdictional reach of national health regulators [13]. These problems would still exist even if certain safeguards were in place, such as limiting registration to licensed healthcare professionals. Indeed, healthcare professionals have been utilized by industry to engage in fraudulent marketing [13,37].

### Recent developments

**Figure 1:**
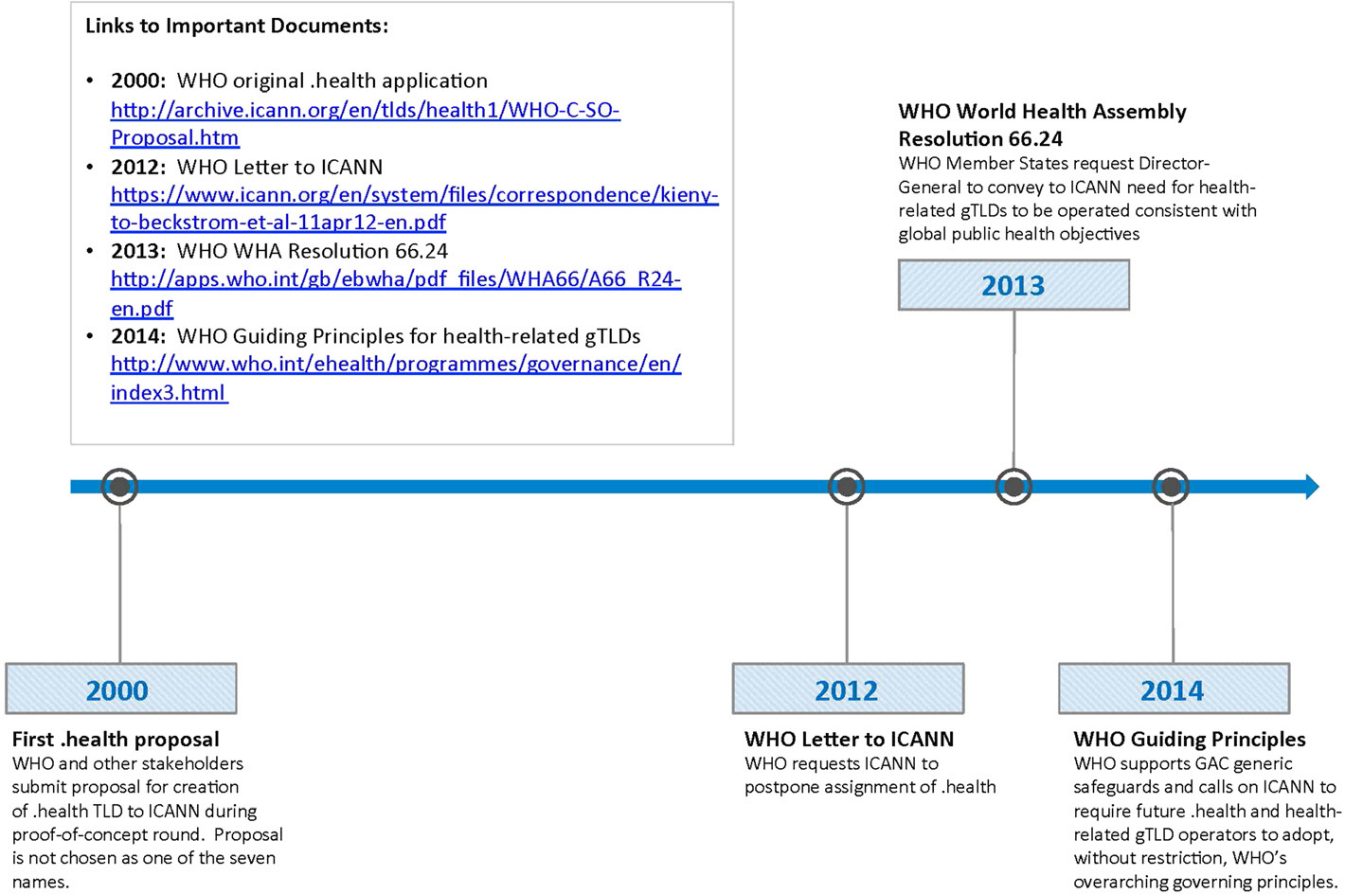
**Timeline for WHO gTLD actions.**

Much of the current situation is explained by WHO’s apparent failure to protect and secure the Health Internet [13]. WHO attempted to acquire and operate the .health domain name as early as 2000, but has been inconsistent and ineffectual in its recent efforts of securing this important piece of future intellectual property on the Health Internet (see Figure 1 for timeline of events) [16,38]. Specifically, WHO failed to submit an application for the .health gTLD in the current ICANN solicitation, thereby taking itself out of the running though it originally sought to be the administrator of the .health TLD [38].

Further, as ICANN’s ongoing processes have exhibited no interest in concerns for public health, WHO’s interventions have become increasingly weaker [1,13,16]. In 2012, WHO specifically requested ICANN indefinitely to “postpone the assignment of the ‘.health’ … until such time as following broad-based consultation of the health community” – a request to which ICANN never responded [39]. Further, in 2013 WHO Member States at the 66th World Health Assembly adopted Resolution 66.24 calling for all health-related gTLDs to be properly governed and operated in a manner consistent with promoting public health in 2013 [40].

However, in May 2014, the WHO Secretariat appears to have contravened Resolution 66.24 of its Member States and backtracked on its position of calling for postponement and consultation. Less than a week before the 67th World Health Assembly began, the WHO Secretariat wrote ICANN again, this time to endorse “safeguards on new gTLDs”, even though the Resolution 66.24 nowhere mentions “safeguards” as an approach that Member States support. The WHO Secretariat offered ICANN this solution without the Member States’ mandate, and despite the fact that the safeguards which ICANN has proposed are hardly protective (e.g., “special safeguards” for trivial and offensive domains like .sucks or .wtf, but no similar protection for health-related domain names) [21].

These moves by the WHO Secretariat – both the backtracking, and the timing just days before the 67th World Health Assembly – suggest that Member States need to be more watchful of the Secretariat in ensuring that it meets its obligations of protecting health-related domains. They must require that WHO Secretariat, as a minimum first step, to follow closely WHO’s program of work outlined in recent gTLD Principles that cover a wide variety of important topics regarding eHealth including: governance and management; transparency; privacy and security; developing codes of conduct for gTLD registrars; individual choice and control over health data; establishing a legal and regulatory framework; and ensuring appropriate delivery of health products and services online and only legitimate activity [41].

However, the newly proposed WHO Principles alone may be inadequate because they are already weak and potentially unenforceable as they function as a “soft law” response (i.e., a set of voluntary codes) that are dependent on existing ICANN processes that so far have failed to recognize the importance of health in the context of Internet governance [1]. In any event, recent decisions by ICANN’s New gTLD Program Committee offer no indication that it will even take into consideration WHO’s proposed principles [1]. Hence, if the WHO Secretariat continues in endorsing ICANN’s proposals, rather than following Resolutions of its Member States, it may prove even weaker as a governance document and reify the need for Member States to demand fulfillment of duly passed World Health Assembly Resolutions.

### Call to action: a moratorium on health domains

The health-related gTLDs should not be governed the same way as other gTLDs. Health is different both in fact and in law. There is an international legal right to health, even if the ICC did not accept that it exists and unreasonably rejected the proposition that the right extends to the availability and accessibility of quality health information [42]. At the same time, more people than ever before are using the Health Internet to seek information and to make behavioral choices [3]. Now is not the time either to compromise this legal right or complicate the factual reality, in favor of profit-making interests merely for the sake of unlimited Internet expansion.

Governing the health-related Internet domains should be a priority for international public health organizations as well as global information technology organizations such as ICANN, WHO, the International Telecommunication Union, the World Summit on the Information Society (“WSIS”), and its establishment of the Internet Governance Forum (“IGF”). Specifically the WSIS and IGF recognize the need for multistakeholder consultations to ensure that both safety and legality prevail on the Internet [11]. Achieving this will require developing norms, in the form of rules and guidelines, establishing decisionmaking processes, transparency policies, and certification/accreditation processes to create a subset of easily identifiable and protected health-related domains for the public interest [13].

However, for this to occur, ICANN must call a halt to its controversial plan to award the remaining health-related gTLDs. We concur with the ICANN Independent Objector, the International Society for Telemedicine & eHealth, the World Medical Association, and the WHO Secretariat (as expressed in its previous positions) in calling for an immediate halt to the ICANN process for health gTLDs [22,39,43,44]. The .health domain, which was imminently scheduled to go to auction, is a very high priority in this regard.

If ICANN fails to appropriately ensure the trust and reliance of applicants that operate the gTLDs it may be appropriate in the future to hold ICANN and awarded registrars legally liable for foreseeable health harms that occur on the Internet. Should ICANN persist in its unregulated process of expanding that namespace, without meaningful safeguards and by ignoring clear warnings from many stakeholders, this would demonstrate a lack of due diligence which the legal system might one day seek to penalize.

Were ICANN to agree to this moratorium, we would recommend the formation of an expert working panel comprised of a diverse set of eHealth stakeholders to constructively discuss the appropriate role and governance of gTLDs to ensure universal access to quality health information online. This includes discussion on consumer privacy and protection, methods of assurance and verification of quality/trusted health information, proactive prevention of online fraud and abuse, regulation of illegal promotion and commercial practices targeted to consumers, and promoting health literacy on the web. The panel could explore more appropriate and alternative policies and governance mechanisms during the time-limited moratorium.

Governance options could include: re-categorizing the .health to a sponsored TLD (“sTLDs”) (explained below); creating new ICANN safeguards to ensure appropriate use; and establishing a permanent multistakeholder compliance monitoring framework for the newly awarded gTLDs [13]. As the WSIS and IGF already have structures for multistakeholder participation in these areas, they may be the most appropriate forums for these needed debates and consultations [11]. We also reject the ICC as a forum for adjudication of these matters, since it is a lobby for industries having a conflict of interest (e.g*.*, pharmaceuticals; health insurers) or often acting in opposition to health (e.g., tobacco, alcohol, fast food, weapons).

These are not exceptional standards, and even in ICANN’s own history, there is precedent for more robust TLD safeguards. In 2003, ICANN created a proposal system and selection criteria for sTLDs [13]. Through that process and previous ICANN expansion rounds, several sTLDs were created including .int, .aero, .coop, .post, .pro, .travel, and .xxx [45]. All these sTLDs require the “sponsor” organization to enforce eligibility of use, ensure transparency and accountability in its operations, and operate the TLD in the interests of the specific community it addresses [45]. As an example, .aero is exclusively used by members of the aviation community, .travel is dedicated to the travel and tourism, .post is restricted to the global postal community with the Universal Postal Union (a U.N. specialized agency) as its sponsor, and .xxx is reserved for the adult entertainment industry [45-47]. We believe that “health” and eHealth are germane to specific communities, and hence requires a responsible steward that will be held accountable to the broader global public health community as well as consumers and patients. Surely if aviation, travel, and adult related TLDs can have restrictions, so should health-related domains.

As the .health gTLD enters its final weeks prior to its published auction in September 2014, the time for the global public health community to coalesce and act is now and with urgency. However, it may unfortunately be too late, as there are unconfirmed reports that ICANN has awarded the .health gTLD to DotHealth LLC through a private settlement prior to the auction [48]. Regardless of the outcome, collectively the public health and broader Internet community need to be vigilant about the need to ensure the reliability and trustworthiness of health information online and take immediate action to ensure the future integrity and proper governance of this important namespace for the Health Internet.
